# Clinicalpathological and prognostic significance of survivin expression in renal cell carcinoma: a meta-analysis

**DOI:** 10.18632/oncotarget.15082

**Published:** 2017-02-04

**Authors:** Zhichen Pu, Guang-Zhen Wu, QiFei Wang

**Affiliations:** ^1^ Department of Urology, The First Affiliated Hospital of Dalian Medical University, Dalian, Liaoning 116011, China; ^2^ State Key Laboratory of Natural Medicines, Key Lab of Drug Metabolism and Pharmacokinetics, China Pharmaceutical University, Nanjing 210009, China

**Keywords:** survivin, renal cell carcinoma, meta-analysis, prognosis

## Abstract

**Background:**

In recent years, survivin expression had been investigated as a prognostic biomarker for renal cell carcinoma (RCC), however, the results were conflicting. This study was aimed to explore the association between survivin expression and clinicalpathological features and the prognostic value for cancer-specific survival (CSS) and overall survival (OS) in RCC.

**Results:**

Eleven studies with 1,697 subjects were included in this meta-analysis. The results showed that survivin expression was associated with higher tumor grade (OR=4.25, 95%CI: 3.04-5.95, p<0.001), advanced tumor stage (OR=3.83, 95%CI: 2.01-7.3, p<0.001) and lymph node metastasis (OR=4.19, 95%CI: 2.34-7.52, p<0.001), but had no association with age, gender or distant metastasis. In addition, survivin expression was also correlated with poor CSS (HR=2.08, 95%CI: 1.07-4.05, p=0.032) and poor OS (HR=2.28, 95%CI: 1.57-3.33, p<0.001).

**Materials and Methods:**

Literature was searched by PubMed, Embase and Web of Science. Hazard ratios (HRs) and 95% confidence intervals (95% CIs) were extracted from eligible studies. Fixed or random effects model were used to calculate pooled HRs and 95%CIs according to heterogeneity.

**Conclusions:**

This study demonstrated that survivin expression was associated with more aggressive clinical features and predicted poor CSS and OS in patients with RCC.

## INTRODUCTION

Renal cell carcinoma (RCC) accounts for over 90% of kidney carcinomas [1]. RCC ranks the seventh and the ninth most prevalent cancer type in men and women, respectively [2]. In 2016, there were estimated 62,700 new cases and 14,240 deaths from RCC in the US [3]. Multiple treatment methods have been applied to treat localized RCC, among which, surgery is the most effective while chemotherapy and radiotherapy have moderate effects. However, approximately 20-30% of all RCC patients are in metastatic RCC (mRCC) state when diagnosed, what is worse, another 20% of patients with localized RCC who receiving surgical resection will have a relapse and progress to mRCC in several years [4]. Unfortunately, mRCC is one of the most treatment-resistant malignant tumors. Therefore, reliable and novel prognostic biomarkers are important to distinguish high risk patients and to improve clinical outcomes of RCC.

Resisting apoptosis is an important feature of cancer, which confers cancer cells the ability to avoid death on various physiologic stresses [5]. Survivin, containing 142 amino acid residues, is a member of the inhibitor of apoptosis (IAP) family proteins [6]. Accumulating evidence showed that survivin counteracted a variety of mediators of apoptosis to block cell death both *in vitro* and *in vivo* [7]. This process in turn facilitates cell proliferation and renders tumor cells resistant to different treatment methods [8]. Survivin was proposed as a promising cancer biomarker [9]. Previous studies have shown that survivin expression predict prognosis in various multiple cancer types including breast cancer [10], gastric cancer [11], colorectal cancer [12] and bladder cancer [13]. A variety of studies have investigated prognostic role of survivin expression in RCC, however, the results were conflicting [14–24]. Therefore, it is necessary to carry out a comprehensive analysis by pooling published data.

In this study, we retrieved relevant literature and extracted data from eligible articles to perform a meta-analysis. We aimed to systematically evaluate the clinicalpathological and prognostic value of survivin expression in patients with RCC.

## RESULTS

### Features of included studies

**Figure 1:**
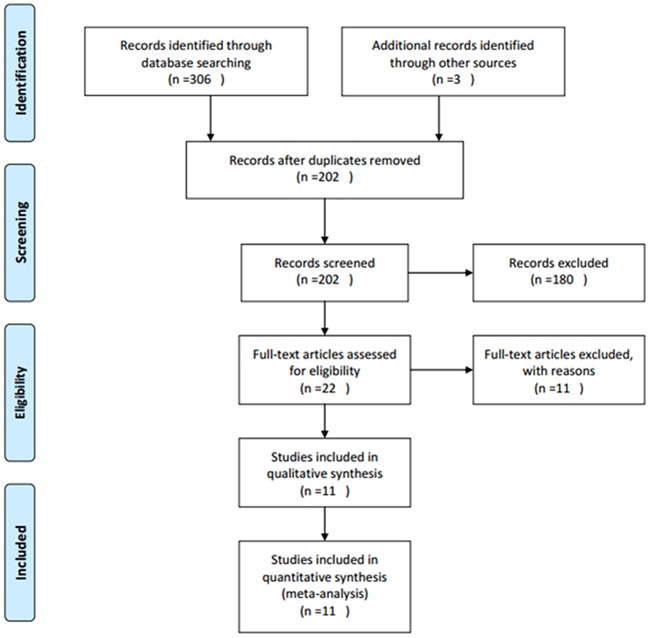
Flow diagram showing process of literature search

A total of 202 studies were identified through systematic literature searching. After title and/or abstract screening, 22 full-text articles were evaluated for eligibility. Then, 11 articles were excluded for: 2 papers were duplicate studies, 1 paper was a comment and 8 studies lacked key information. At last, 11 studies [14–24] published from 2007 to 2015 with 1,697 patients were included in meta-analysis. The literature selection process was shown in Figure 1. All studies were retrospective study design and detected survivin expression using IHC. Five studies [14, 18, 21, 23, 24] were from Asian countries and six studies [15–17, 19, 20, 22] were from western countries. The sample size ranged from 42 to 634. The basic features of these studies were shown in Table 1.

**Table 1:** Basic characteristics of included studies

Study	Year	Country	Ethnicity	Cases	Gender(M/F)	Method	Cut-off level	Survivin (+)N(%)	Histological type	Survival analysis
**Byun**	2007	Korea	Asian	85	63/22	IHC	>10% staining	67(79)	RCC	OS, RFS
**Parker**	2008	USA	Caucasian	310	185/125	IHC	>2% staining	105(33.9)	ccRCC	CSS
**Zamparese**	2008	Italy	Caucasian	49	37/12	IHC	>5% staining	40(81.6)	RCC	CSS
**Parker**	2009	USA	Caucasian	634	413/221	IHC	>15% staining	NA	RCC	CSS
**Lei**	2010	China	Asian	75	36/39	IHC	>10% staining	40(53.3)	RCC	OS
**Baytekin**	2011	Turkey	Caucasian	104	NA	IHC	>5% staining	24(23.1)	RCC	CSS
**Dornbusch**	2013	Germany	Caucasian	42	29/13	IHC	NA	20(47.6)	mRCC	OS
**Liu**	2014	China	Asian	90	52/38	IHC	>20% staining	74(82.2)	ccRCC	CSS
**Weber**	2014	Germany	Caucasian	145	88/57	IHC	NA	NA	ccRCC	CSS
**Lu**	2015	China	Asian	98	63/35	IHC	>10% staining	51(52)	ccRCC	OS
**Shi**	2015	China	Asian	65	37/28	IHC	>5% staining	47(72.3)	ccRCC	OS

IHC: immunohistochemistry; NA: not available; RCC: renal cell carcinoma; ccRCC: clear cell renal cell carcinoma; mRCC: metastatic renal cell carcinoma; OS: overall survival; CSS: cancer-specific survival; RFS: recurrence-free survival.

### Clinicopathological parameters and survivin expression

**Figure 2:**
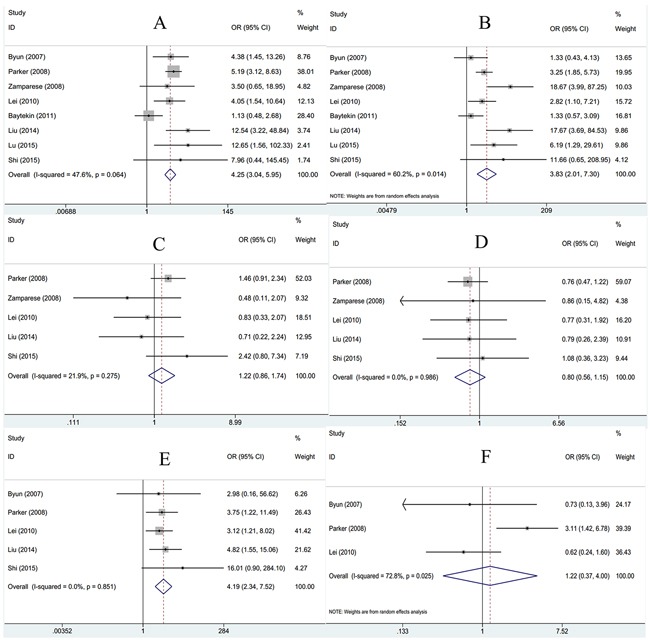
Association between survivin expression and **A**. tumor grade; **B**. tumor stage; **C**. age; **D**. gender; **E**. lymph node metastasis and **F**. distant metastasis in RCC.

To disclose the significance of survivin in pathological diagnosis, we explored the correlation between survivin expression and clinicopathological characteristics. Data of tumor grade, tumor stage, age, gender, lymph node metastasis and distant metastasis were extracted and then pooled OR and 95% CI were computed. As shown in Figure 2, tumor grade (G3+G4, +) was associated with survivin expression (n=8, OR=4.25, 95%CI: 3.04-5.95, p<0.001) in fixed-effects model analysis. Furthermore, there were association between tumor stage (III+IV,+) (n=8, OR=3.83, 95%CI: 2.01-7.3, p<0.001; I^2^=60.2%, P_heterogeneity_=0.014), lymph node metastasis (yes, +)(n=5, OR=4.19, 95%CI: 2.34-7.52, p<0.001; I^2^=0, P_heterogeneity_=0.851) and survivin expression. However, survivin expression had no association with age (n=5, OR=1.22, 95%CI: 0.86-1.74, p=0.271), gender (n=5, OR=0.8, 95%CI: 0.56-1.15, p=0.224) or distant metastasis (n=3, OR=1.22, 95%CI: 0.37-4, p=0.748). Taken together, the results demonstrated survivin expression in RCC patients could be considered as a significant biomarker for diagnosis of patients with higher grade, advanced stage and lymph node metastasis.

### Prognostic value of survivin expression for CSS and OS

**Figure 3:**
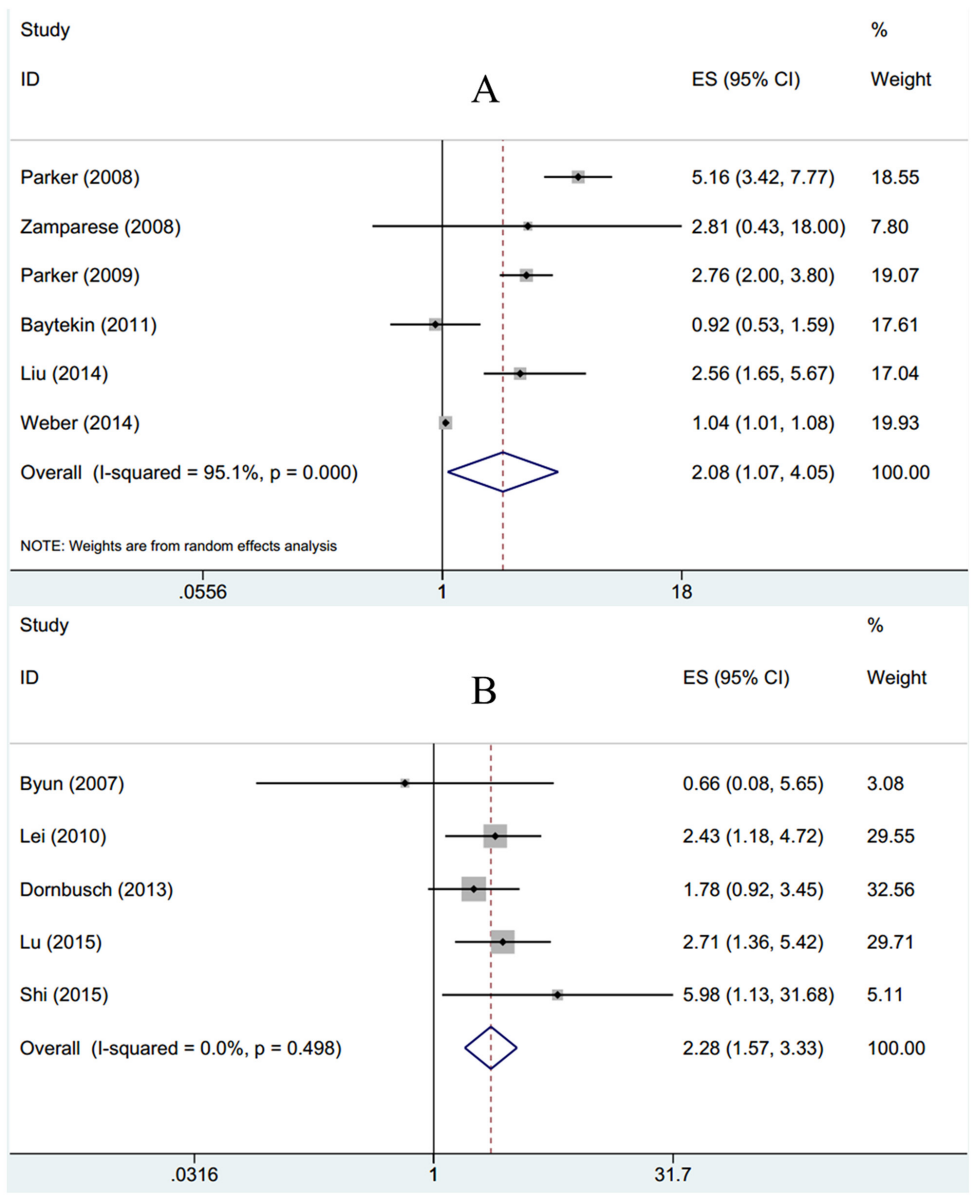
Forest plots to assess effect of survivin on **A**. CSS and **B**. OS in RCC.

To further estimate the association between survivin expression and prognosis for CSS and OS in RCC patients, combined HRs and 95%CIs were calculated. As shown in Table 2 and Figure 3, survivin expression was associated with poor CSS according to pooled data (n=6, HR=2.08, 95%CI: 1.07-4.05, p=0.032; I^2^=95.1%, P_heterogeneity_<0.001, Table 2, Figure 3). Subgroup analysis indicated that survivin expression had no association with Caucasian patients, in RCC or in clear cell renal cell carcinoma (ccRCC) histological type (Table 2). Meanwhile, survivin expression was shown to be related with poor OS generally and the HR was 2.28 with 95%CI: 1.57-3.33, p<0.001, in addition, there was no heterogeneity (I^2^=0, P_heterogeneity_=0.498). Through subgroup analysis, the results showed that survivin expression still had association with Asian patients (n=5, HR=2.57, 95%CI: 1.63-4.07, p<0.001), in RCC (n=2, HR=2.15, 95%CI: 1.11-4.16, p=0.023) and in ccRCC (n=2, HR=3.04, 95%CI: 1.61-5.76, p=0.001).

**Table 2:** Pooled HRs and 95% CIs in meta-analysis for CSS and OS

Variable	Studies (n)	Heterogeneity		HR	95% CI	p	Effects model
		I^2^(%)	P_heterogeneity_				
**CSS**							
**Overall**	6	95.1	<0.001	2.08	1.07-4.05	0.032	Random
**Subgroup1:ethnicity**							
**Caucasian**	5	95.7	<0.001	1.99	0.94-4.23	0.072	Random
**Asian**	1	-	-	2.56	1.38-4.75	0.003	-
**Subgroup2: histology**							
**RCC**	3	82.9	0.003	1.78	0.72-4.43	0.214	Random
**ccRCC**	3	97	<0.001	2.36	0.75-7.44	0.142	Random
**OS**							
**Overall**	5	0	0.498	2.28	1.57-3.33	<0.001	Fixed
**Subgroup1:ethnicity**							
**Caucasian**	1	-	-	1.78	0.92-3.45	0.086	-
**Asian**	4	0	0.463	2.57	1.63-4.07	<0.001	Fixed
**Subgroup2: histology**							
**RCC**	2	22	0.258	2.15	1.11-4.16	0.023	Fixed
**ccRCC**	2	0	0.39	3.04	1.61-5.76	0.001	Fixed
**mRCC**	1	-	-	1.78	0.92-3.45	0.086	-

### Publication bias

**Figure 4:**
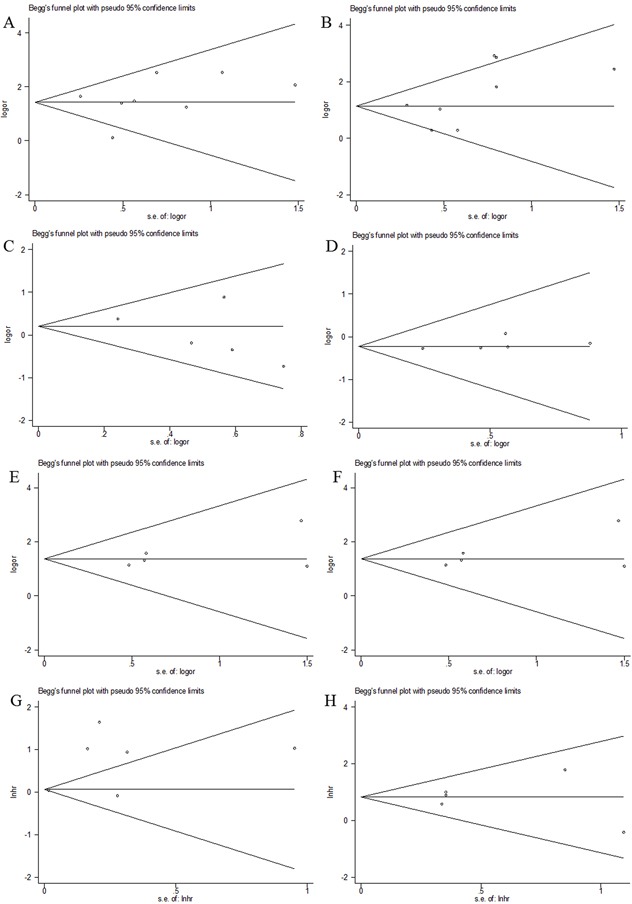
Funnel plots evaluating possible publication bias for **A**. tumor grade; **B**. tumor stage; **C**. age; **D**. gender; **E**. lymph node metastasis; **F**. distant metastasis; **G**. CSS and **H**. OS.

Funnel plots for meta-analysis of survivin expression and clinical features as well as CSS and OS were shown in Figure 4. The funnel plots for all analysis were symmetric, indicating no obvious publication bias (Begg's p: tumor grade: p=0.711; tumor stage: p=0.266; age: p=0.221; gender: p=0.221; lymph node metastasis: p=0.462; distant metastasis: p=1; CSS: p=0.851; OS: p=1; Figure 4).

## DISCUSSION

Survivin has the ability to inhibit apoptosis and it is also needed for cell division [25]. In several animal models, downregulation of survivin was shown to suppress tumor growth and survivin was validated as a cancer therapeutic target [6, 26]. In recent years, emerging data suggested that survivin expression can serve as a promising biomarker for prognostication in various tumors, including RCC [15–17, 23, 24]. The conflicting results from different research groups promote us to perform this meta-analysis. In the present study, based on results form 11 studies with 1,697 subjects, the data showed that survivin expression was associated with higher tumor grade, advanced tumor stage and lymph node metastasis. Moreover, the results for prognosis analysis also indicated that survivin expression was a predictor for shortened CSS and OS. These results suggested that survivin detection was feasible for tumor aggressiveness evaluation and tumor staging. Survivin could be recommended as a valuable risk factor for RCC diagnosis and prognosis. To our knowledge, this is the first study investigating the prognostic role of survivin for RCC patients by the analytic approach of meta-analysis.

Survivin is a structurally unique member of IAP family. Survivin has been found to suppress apoptosis via inactivation of caspases [27]. Besides apoptosis, survivin also takes part in other physiological procedures such as cellular stress response as well as surveillance checkpoints [28, 29]. Survivin is highly expressed in fetal tissues and in various human solid tumors. Its multiple functions could facilitate tumor growth and progression in different aspects. Survivin can mediate mitotic progression so as to promote cell division [30]. In addition, survivin is also involved in angiogenesis and its expression is upregulated when exposed to culture with angiogenic factors such as VEGF [31]. This may explain the positive correlation between survivin expression and lymph node metastasis in this meta-analysis. Taken together, current evidence suggests that survivin plays a pivotal role in cancer formation and progression.

The results in the present meta-analysis demonstrated that survivin was a predictor for poor prognosis in RCC, which was in line with conclusions from other solid and hematological cancer types including breast cancer [10], non-Hodgkin's lymphoma [32], non-small cell lung cancer [33], glioma [34] and gastric cancer [35]. In addition, we also analyzed the association between survivin expression and clinical factors in RCC and we found that survivin had positive relationship with higher tumor grade and tumor stage. These risk factors are well established in clinical practice and are often used to aid therapeutic regimen selection. The correlation between survivin and the factors revealed that survivin had potential to be adopted as a dichotomous biomarker. The present study is the first meta-analysis systematically evaluating the prognostic value of survivin expression in RCC to date.

Several limitations need to be addressed. First, significant heterogeneity was detected for several parameters, although we picked random-effect model or fixed-effect model according to heterogeneity, it still existed due to the difference in included studies. Second, the scale of RCC patients was relatively small; therefore, large scale studies are needed to conceive more reliable results. Third, subgroup analyses for Asian patients in CSS and Caucasian patients and mRCC in OS were not actually conducted because only one study was included in each of these groups. Thus the results concerning these subgroup patients need to be completed in further studies.

In conclusion, this meta-analysis showed that survivin expression was associated with more aggressive clinical features and predicted poor CSS and OS in patients with RCC. Due to limitations in this study, large scale studies with more complete patients’ types are needed to verify our results.

## MATERIALS AND METHODS

### Literature search

A comprehensive literature search was conducted in platforms of PubMed, Embase and Web of Science. The last search was performed on April 2016. The MeSH terms and free words adopted were as follows: “survivin”, “baculoviral inhibitor of apoptosis repeat containing 5”, “BIRC5”, “renal cancer”, “kidney cancer”, “renal carcinoma” and “renal cell carcinoma” and their combinations. The reference lists of previous relevant reviews were also manually checked to find additional publications of interest. The language of publications was restrained to English.

### Inclusion and exclusion criteria

The following inclusion criteria were used to select eligible studies: (i) the diagnosis of RCC was pathologically confirmed; (ii) the prognostic value of survivin expression for overall survival (OS), cancer-specific survival (CSS) and/or recurrence-free survival (RFS) were reported; (iii) the expression of survivin was tested by immunohistochemistry (IHC) method; (iv) hazard ratios (HRs) and their 95% confidence intervals (95% CIs) for survival analysis were reported in text or could be computed from given data; (v) if more than one articles from one patients cohort were identified, the most complete one was selected. The exclusion criteria were: (i) abstract, review, case report or comment letter; (ii) animal studies; (iii) duplicate publications; (iv) published not in English.

### Data extraction

Two independent reviewers using a standardized form extracted relevant data from eligible studies. The needed information was: first author's name, year of publication, origin country of the study, cases, cut-off levels, histology and survival end point. Discrepancies were discussed until reaching a consensus.

### Statistical analysis

Data were analyzed by using Stata 12.0 (StataCorp LP, College Station, TX, USA). Odds ratio (OR) and 95% CI were used to present the associations between clinical factors and survivin expression. Hazard ratio (HR) with a 95% confidence interval was computed to reveal the correlation between survivin expression and prognosis (CSS and OS). If statistical variables were reported in text, then we extracted them directly, otherwise, data was calculated according to the method provided by Tierney [36]. Heterogeneity among studies was examined using Chi-square based Q test in which I^2^ indicates level of heterogeneity. I^2^<50% or P_heterogeneity_>0.1 represents low heterogeneity, in this case, a fixed effects model (Mantel-Haenszel method) was used, otherwise, a random effects model (Der Simonian and Laird method) was picked. Subgroup analysis was performed for CSS and OS analysis. Publication bias was examined by using Begg's funnel plot. P<0.05 was considered as statistically significant.
